# Crystal structure of the African swine fever virus structural protein p35 reveals its role for core shell assembly

**DOI:** 10.1007/s13238-020-00730-w

**Published:** 2020-06-09

**Authors:** Guobang Li, Dan Fu, Guangshun Zhang, Dongming Zhao, Mingyu Li, Xue Geng, Dongdong Sun, Yuhui Wang, Cheng Chen, Peng Jiao, Lin Cao, Yu Guo, Zihe Rao

**Affiliations:** 1 State Key Laboratory of Medicinal Chemical Biology and College of Pharmacy, Nankai University, Tianjin, 300350 China; 2 State Key Laboratory of Veterinary Biotechnology, Harbin Veterinary Research Institute, Chinese Academy of Agricultural Sciences, Harbin, 150001 China; 3 School of Life Sciences, Tianjin University, Tianjin, 300071 China

**Dear Editor,**

The African swine fever (ASF) is a highly contagious hemorrhagic and lethal disease in domestic pigs. The ASF outbreaks of in many Asia countries, including China, Vietnam, Mongolia, and South Korea, have posed a huge threat to the pig industry. In the attempt to stop ASF from spreading further in Mainland China, more than 10 million pigs have been culled since August 2018. The causative agent for ASF is a DNA virus, African swine fever virus (ASFV). Although this deadly disease has been reported in Kenya nearly one hundred years ago, there has been no vaccine for protection from ASFV infection or effective treatments to cure ASF until now.

ASFV, the only species of *Asfarviridae*, is a large, enveloped virus with a regular structure. The DNA genome of ASFV vary in length from approximately 170 to 193 kbp and encodes 151 to 167 proteins depending on the different viral isolates (Dixon et al., 2013). The ASFV virion comprises more than 50 polypeptides and possesses a multilayers structure: the external envelop, the icosahedral protein capsid, the inner membrane, the core shell, and the genome-containing nucleoid (Andrés et al., 1997). Since the progeny virus proliferates in the eukaryotic cytoplasm, the ASFV is categorized into the nucleocytoplasmic large DNA viruses (NCLDVs) family. The external lipids envelop of the ASFV virus is finally obtained during the virus assembly process, which is originated from the host cell plasma membrane. Beneath the external lipids envelop is the icosahedral protein capsid. This layer contains many components, including the trimer major capsid protein p72 and other minor capsid proteins (M1249L, p17, p49, and H240R, etc.) (Wang et al., 2019). The structure features of ASFV p72 are very similar to some NCLDVs MCP (major capsid protein), mainly displaying a double jelly-roll fold. The inner membrane layer, locating between the icosahedral protein capsid and core shell, is derived from the endoplasmic reticulum. The core shell is a thick protein layer, with approximately 30 nm in thickness. Research has shown that the mature proteins, p150, p37, p34, p14, and p5 derived from polyprotein pp220 and p35, p15, and p8 derived from polyprotein pp62, are the major components of the core shell (Andres et al., 2002).

The recent researches on ASFV virion by Cryo-EM method depict the architecture of the icosahedral protein capsid which contains the major capsid protein p72 and some other minor capsid proteins (M1249L, p17, p49, and H240R) (Liu et al., 2019b; Wang et al., 2019; Andres et al., 2020). However, the core shell is only reconstructed to 9 Å, containing 1,806-blade propeller-like capsomers with a central channel and 12 starfish-like pentons surrounded by 10 antennae, and yielding a *T* = 19 icosahedral lattice (Wang et al., 2019). Previous work has also proved that both the extracellular and intracellular ASFV particles are infectious, which indicates that the external membrane is not strictly necessary for infectivity (Andrés et al., 2002). The structural and functional information for core shell remains elusive in NCLDVs family, atomic-resolution structure of the whole core shell and the individual component will greatly aid the understanding of ASFV assembly mechanism.

Herein, we present the crystal structure of ASFV structural protein p35 (304 amino acid residues) and explore its potential role in core shell assembly. The polyprotein pp62 produces three mature structural proteins by the cleavage of viral protease pS273R, give rise to mature product p15, p35 and p8 (Fig. 1A). The full-length p35 was expressed in *Drosophila* S2 cells with a hexahistidine tag at its C terminus, and purified by immobilized metal ion affinity chromatography (IMAC) and size exclusion chromatography (SEC). The SEC result shows that ASFV p35 may exist multiple forms in solution, with the monomer as predominant peak (Fig. 1B). To further confirm the p35 oligomers’ existence, the AUC (analytical ultracentrifugation) experiment was performed under physiological neutral pH or acidic conditions. The AUC results under neutral pH condition (7.0) indicate that the recombinant p35 predominantly exist as the monomer, with very little dimers or higher oligomers in solution (Fig. 1C). Previous works on virus matrix protein in *orthomyxovirus* have shown that solution pH affects the intermolecular interaction for virus matrix protein, with dimers observed at low pH (Sha and Luo, 1997) and monomers at neutral pH condition (Arzt et al., 2001). The native gel result from different pH gradient indicates a similar transition for ASFV p35 (Fig. 1D), which implies the possibility of conformation change of p35 protein during the virus entry and uncoating process in ASFV infectious cycle.

**Figure 1:**
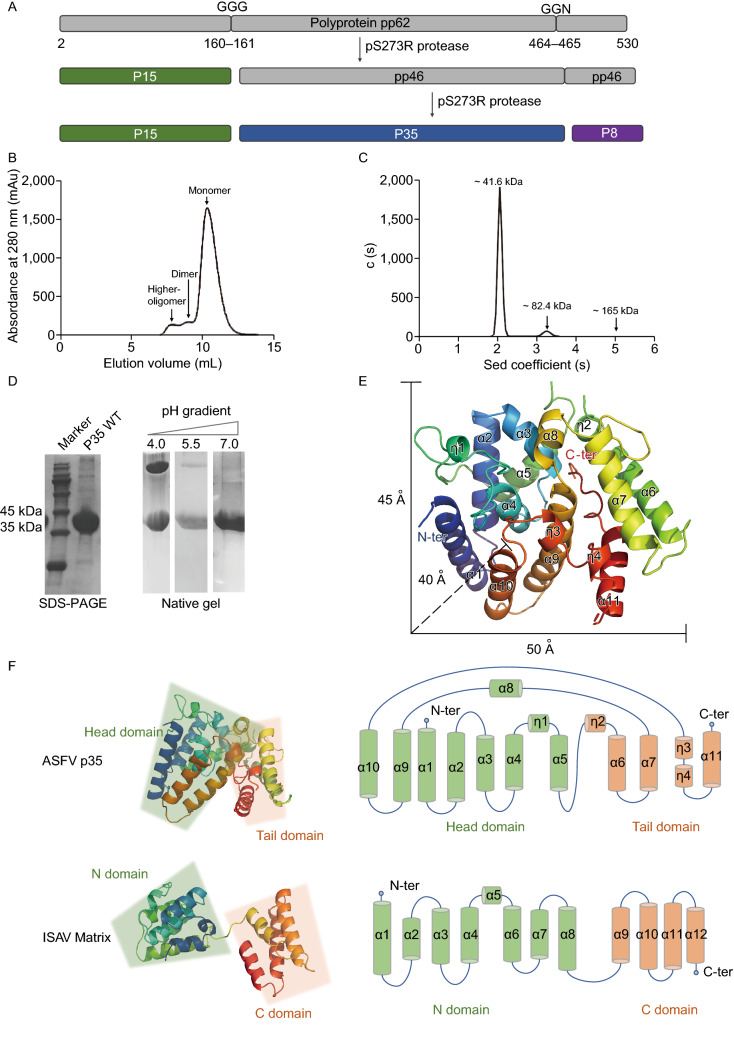
**The overall structure of ASFV p35 protein**. (A) Schematic representation of polyprotein pp62 catalyzed into mature structural proteins: p8, p35, and p15. The precursor pp62 and intermediate precursor pp46 are coloured in grey; the mature proteins p15, p35, and p8 are coloured in green, blue, and purple, respectively. The cleavage sites “GGG” and “GGN” are labelled on the top. (B) Purification of ASFV p35. The protein is purified over a superdex75 16/60 size-exclusion column. The black line indicates the absorbance at UV280 nm. Three peaks are corresponding to monomer, dimer and higher oligomer. (C) The calculated molecular weights corresponding to each peak in AUC are labelled above the curve. Sed stands for sedimentation. (D) SDS-PAGE gel of ASFV p35 from the fraction in the monomer peak (left), and the native gel in different solution pH (right). (E) The crystal structure of ASFV p35 is shown in cartoon. The secondary structure elements are numbered consecutively with the N terminus and C terminus labelled. The size of the ASFV p35 protein is labelled. (F) Structures of ASFV p35 protein and IASV Matrix are shown in cartoon. The topology of two structure are also presented; secondary structure elements are numbered consecutively

Two different shapes of crystal are obtained by the vapour-diffusion method. The rode-like crystals appear in the condition containing 0.2 mol/L ammonium acetate, 0.01 mol/L magnesium acetate tetrahydrate, 0.05 mol/L sodium cacodylate trihydrate pH 6.5, 30% PEG 8000, which is subject to heavy atom screening and soaking. While the high-resolution data were collected by the cube-like crystal grows in 0.1 mol/L BIS-TRIS pH 5.0, 25% *w*/*v* PEG 3350. Due to the lack of a predicated homology model, the crystal structure of p35 is finally determined using single-wavelength anomalous diffraction (SAD) by a mercury (II) derivative and refined to 2.1  Å with a *R*_factor_ and *R*_free_ of 20.3% and 23.0% (Table S1). The rod-like crystal grows in relative higher pH solution belongs to space group *P*2_1_ with one p35 molecule in one asymmetric unit, in contrast to the cube-like crystals with larger unit cell and containing two p35 molecules in one asymmetric unit. In the final model, residues D1-Y11, D128-D138, G265-I273 and E299-N304 of Molecular A and B are not visible due to the lack of interpretable electron density data, suggesting their high structural flexibility.

The ASFV p35 is a compact structure composed of eleven α-helices and four 3_10_-helices (Fig. 1E). Homology search by the DALI server gives no structural homology with any identified proteins, indicating ASFV p35 as a completely novel fold structure. The structure of p35 can be described as two lobes of helix bundles, termed as a larger “head domain” and a relatively smaller “tail domain”, respectively (Fig. 1F). The head domain is a helix bundle with the central α4 surrounded by α1, α2, α3, α9 and α10, mainly stabilized by hydrophobic force, and further packed by α5, α8, and η1. The tail domain is a helix bundle composed by α6, α7, α11 and η4 (Fig. 1E and 1F). This helix bundle-like structure is very common for RNA virus matrix protein, for instance, in the *orthomyxovirus* family. We compare the fold pattern of p35 with the ISAV (infectious salmon anemia virus) matrix protein, which is the only virus that the full-length matrix structure has ever been reported in *orthomyxovirus* to date. The polymerized matrix proteins M1 of ISAV are underneath the viral envelope to enforce the shape and structural integrity (Zhang et al., 2017). Similarly, ISAV matrix M1 is composed of a helix bundle consisting of twelve α-helices, and can be divided into two separate domains: termed as “N-domain” and “C-domain”. The “N-domain” connects with the “C-domain” of the ISAV matrix by one flexible loop, which located between α8-helix and α9-helix (Fig. 1F). In contrast, for ASFV p35, the two lobes are linked by three loops, rendering it as a much more compact structure (Fig. 1F).

Since different oligomerization states have been observed during the purification process, and the crystals grow in neutral or acidic conditions also result in two types of crystal forms with different molecule packing mode, we next investigate the molecule contacting interface via PISA (protein interfaces, structures and assemblies) analysis software. The result shows that the accessible surface area buried in the interface is 485.2 Å^2^, only 4% of the overall surface, mainly via hydrophobic force and some electrostatic interactions, contributed by N180, V176, N173, F205, W206, I253, N251, and N254 from Molecule A, P98, T99, L106, F50, L53, N54, H55, and P56 from Molecule B (Fig. 2A). The PISA result strongly suggests that the interaction within two molecules is highly possible due to crystal packing, but not reflecting the authentic oligomerization state during ASFV infectious cycle. Future work on ASFV core shell conformation change during virus assembly and budding will elucidate this question.

**Figure 2:**
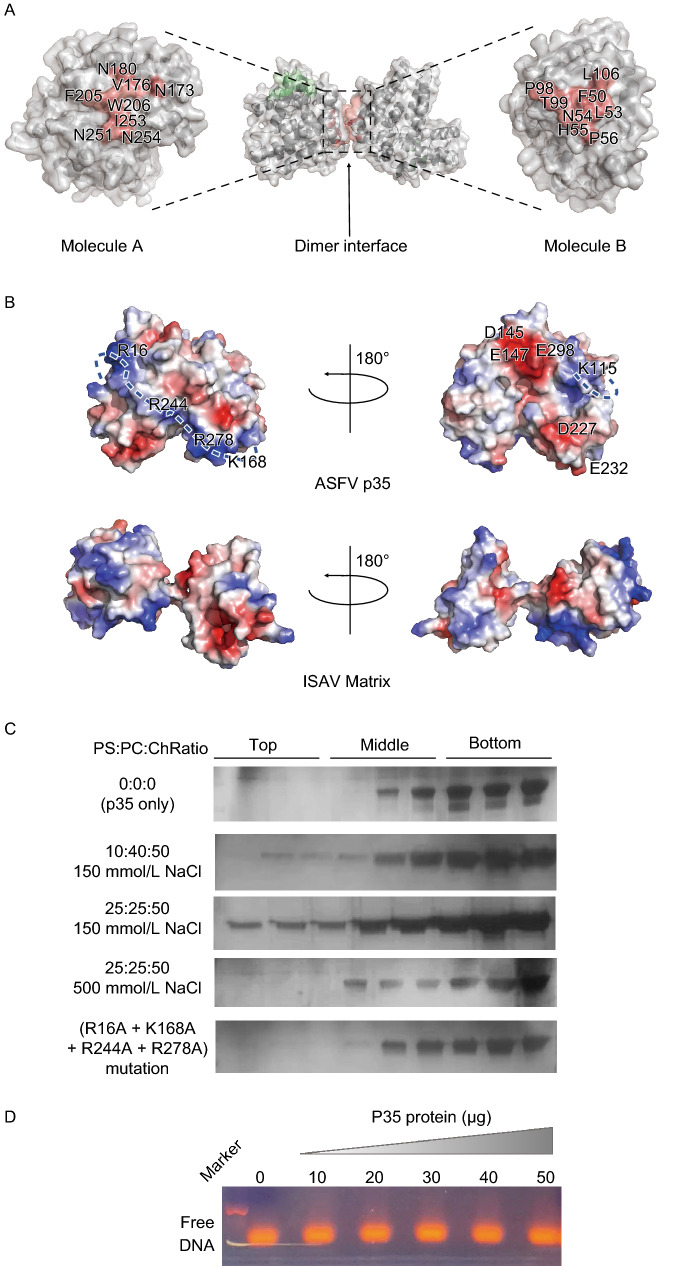
**The characteristics of ASFV p35 protein for membrane-association and DNA binding**. (A) Surface diagram of ASFV p35 protein. Molecule A with Molecule B forms a dimer through hydrophobic force and some electrostatic interactions, the residues on the interface are labelled. The regions within the dimer interface are coloured salmon, and the interface on the other side of the molecule A is coloured green. (B) Electrostatic properties of ASFV p35 and ISAV matrix (two orientations, rotated by 180°). The positive and negative charges are coloured from blue to red with limits ±5 kT/e. The key basic residues and acidic residues are labelled on the p35 protein surface. The continuous positive charges bulge on the p35 surface are indicated by a blue dotted line. (C) ASFV p35 membrane association by liposome flotation. ASFV p35 protein and mutant are incubated overnight with liposomes under different conditions and floated on an *Accudenz* discontinuous gradient. Top, Middle, and Bottom fractions of the discontinuous gradient were analyzed by SDS-PAGE and stained with Rapid Silver Staining Kit. (D) EMSA assay of ASFV p35 binding to dsDNA derived from ASFV genome

Numerous studies on virus matrix protein have indicated its critical role for membrane association primarily mediated by a positively charged patch from the protein surface (Rao et al., 1995; Ruigrok et al., 2000; Zhang et al., 2017). For ASFV p35, there are two charged patches in p35 protein surface: a continuous positive charged bulge composed of residues R16, R244, R278, and K168; and a negatively charged patch, which contains the residues D145, E147, E298, D227, and E232 (Fig. 2B). To test the lipid membrane binding ability of p35, we perform the liposome floatation experiments, following a previously described protocol with some modification (Ruigrok et al., 2000). The result shows the p35 proteins cannot be detected in top fractions without liposome but can be detected in top fractions after incubation with liposome in different lipid compositions (Fig. 2C). Moreover, p35 shows strong preference for PS (phosphatidylserine, 1, 2-dioleyl-sn-glycero-3-phospho-L-serine) over PC (phosphatidylcholine, 1-palmitoyl-2-oleyl-sn-glycero-3-phosphocholine), when the ratio of PS to PC is increased from 10:40 to 25:25, the p35 protein in the top fraction also increasing significantly. Meanwhile, the high ionic strength (500 mmol/L NaCl) prevents the lipid-binding, indicating that the ASFV p35 binding to lipids is largely mediated by electrostatic interactions (Fig. 2C). The mutants of ASFV p35 (R16A + K168A + R244A + R278A) completely abolish the lipid-binding activity (Fig. 2C), further confirm the importance of the positively charged patch for facilitating core shell binding with the inner membrane. This phenomenon is also observed for ISAV matrix protein (Zhang et al., 2017). Phosphatidylserine is universally present in the inner leaflet of the plasma membrane in all multicellular organisms ranging from mammals to nematodes (Kloditz et al., 2017; Zhang et al., 2017). It is highly possible that the interaction between ASFV p35 and phosphatidylserine may facilitate the recruitment of p35 and other core shell components to the plasma membrane. Besides, electrophoretic mobility shift assay (EMSA) was carried out to test the possibility of ASFV p35 proteins binding with dsDNA (25 base pairs in length, derives from the ASFV genome). No shift for DNA probes has been observed even at a high concentration of ASFV p35 proteins (Fig. 2D). This result indicates that recombinant ASFV p35 in this work does not possess DNA binding activity.

Recent studies investigate the extracellular ASFV multilayers complex structure by Cryo-EM (Liu et al., 2019b; Wang et al., 2019; Andres et al., 2020). The structure of major capsid protein p72 and the architecture of outer regular icosahedron capsid have been studied clearly (Liu et al., 2019a; Liu et al., 2019b; Wang et al., 2019; Andres et al., 2020). However, the core shell architecture or components’ structure remains elusive. The thick core shell layer account for about one-third mass of the ASFV virus, and is composed of several mature proteins derived from polyprotein pp220 and pp62 (Andrés et al., 2002). In our study, we present the crystal structure of p35, which is the cleavage product protein derived from the polyprotein pp62. The recombinant p35 adopts a novel fold structure, shares no homology with any reported structure, and also gives rise to an interesting question about the origin and evolution pattern of ASFV. Moreover, we confirmed the positively charged patch plays an important role in lipid binding activity. It is worth mentioning that the role of the negatively charged patch on the other side of the positively charged patch remains elusive. One possible answer for this is, the negatively charged patch together with other regions may serve as an interface to facilitate the interaction with multiple structural proteins. Membrane-associated proteins such as p35 may serve as a docking scaffold for the recruitment of host membrane and other components in the core shell during the virus assembly process and further stabilize the mature virion. We anticipate more work to illustrate the assembly and working mechanism of ASFV core shell.

## Electronic supplementary material

Below is the link to the electronic supplementary material.

Supplementary material 1 (PDF 248 kb)
