# Supplementary material for: Crystal structure of the African swine fever virus structural protein p35 reveals its role for core shell assembly
Source: Protein Cell. 2020 Jun 9;11(8):600–5. doi: 10.1007/s13238-020-00730-w (PMC7381542; doi:10.1007/s13238-020-00730-w)
Supplement: Supplementary file 1 — Supplementary material 1 (PDF 248 kb) [file 13238_2020_730_MOESM1_ESM.pdf]

Supplementary Information

Crystal structure of the African swine fever virus structural protein p35 reveals its role for core shell assembly

Guobang Li*, Dan Fu*, Wei Zhang*, Dongming Zhao, Mingyu Li, Xue Geng, Dongdong Sun, Yuhui Wang, Cheng Chen, Peng Jiao, Lin Cao [#], Yu Guo[#], Zihe Rao

State Key Laboratory of Medicinal Chemical Biology and College of Pharmacy, Nankai University, Tianjin 300350, People's Republic of China
State Key Laboratory of Veterinary Biotechnology, Harbin Veterinary Research Institute, Chinese Academy of Agricultural Sciences, Harbin 150001, People's Republic of China
School of Life Sciences, Tianjin University, Tianjin 300071, People's Republic of China

* These authors contributed equally to this paper;
[#] Correspondence: guoyu@nankai.edu.cn (Y.G), caolinzz@mail.nankai.edu.cn (L.C)

Contents

MATERIALS AND METHODS

Supplementary Table 1

MATERIALS AND METHODS

Expression and purification. A synthetic gene coding for the ASFV p35 (GenBank ID: MK128995.1) codon-optimized for Drosophila expression, was

initially inserted into a modified pMT/BiP plasmid (Invitrogen), in frame with the BiP signal sequence encoding segment upstream, and fused downstream with C terminal hexahistidine tag. The resulting plasmids were used to transfect *Drosophila* S2 cells together with the pCoHygro plasmid. Stable cell lines were selected and maintained in serum-free ESF921 medium containing 15 μg/mL hygromycin B. Cells were grown to 4.0×10^6 cells ml^{-1} in ESF921 medium, and expression was induced with 1 mM $CuSO_4$. After 4-6 days, the supernatant of the S2 cells was harvested. The recombinant ASFV p35 proteins were purified preliminarily on a Ni-NTA affinity column and further purified by Superdex 75 16/60 size-exclusion column in 20 mM HEPES, 150 mM NaCl, pH7.5. Fractions were concentrated to 10 mg/ml for crystallization.

Native-PAGE. Natural polyacrylamide gel electrophoresis without SDS can keep biological macromolecules in their natural shape and charge during electrophoresis (Reifschneider et al., 2006). To further understand the oligomer states of p35, Native-PAGE was used to run the protein under a range of pH 5.0, 6.0, 7.0, respectively. 100 V constant voltage for about 20min, the indicator enters the concentrated gel; change the 160V constant voltage, when the indicator moves to the bottom of the rubber plate stop the electrophoresis. The native gels were stained with Coomassie Brilliant Blue.

Crystallization. The crystallization screenings were carried out by the sitting-drop vapour-diffusion method using commercially available solutions (Hampton Research) at 289K. The protein of p35 and reservoir solutions were mixed in a ratio of 1:1, and all conditions were equilibrated against 100 μl of reservoir solution in a 48-well format plate. The cube-like crystals grew in a reservoir solution condition (0.1MBIS-Tris pH 5.0, 25% W/V Polyethylene glycol 3350) and the rode-like crystals grew in the presence of 0.2M Ammonium acetate, 0.01 M Magnesium acetate tetrahydrate, 0.05M Sodium cacodylate trihydrate pH 7.0, 30% W/V Polyethylene glycol 8000 in about one week. The rode-like crystals were soaked reservoir solution

adding 3 mM K_2HgI_4 for abnormal signal data collection. Crystals were cryoprotected in crystallization solution with 30% PEG4000 and cooled in a dry nitrogen stream at 100 K for X-ray data collection.

X-ray data collection and structure determination. Diffraction data for the native crystal of ASFV p35 was collected on beamline BL17U1 at SSRF (Shanghai Synchrotron Radiation Facility. Anomalous diffraction data for mercury derivative was collected on beamline BL19U1 at SSRF. All datasets were indexed, integrated, and scaled using the HKL3000 package. Heavy atom searching, initial phase calculations, and density modifications were performed with PHENIX (Adams et al., 2002). A model was manually built into the modified experimental electron density using COOT (Emsley and Cowtan, 2004) and further refined in PHENIX. Model geometry was verified using the program MolProbity (Chen et al., 2009). Structural figures were drawn using the program PyMOL (http://www.pymol.org).

Analytical ultracentrifugation (AUC) assay. AUC experiments were performed at 4℃ in a Beckman Optima XL-I, operating under velocity sedimentation (SV) mode using an AN-50 Ti rotor with two-channel charcoal-filled centrepieces. Sedimentation velocity experiments were performed at 40,000 rpm loading 1 mg/ml protein. Data were collected at 280 nm in a continuous mode and fitted with SEDFIT.

Mutant protein production. The site-directed mutations (R16+K168+R244+R278) of p35 were generated by using the Fast Mutagenesis System (TransGen Biotech, China). The sequence of all the mutation constructions was verified by DNA sequencing (GENGWIZ, China). The mutant protein was expressed and purified following the procedure of the wild-type protein. The AUC elution profiles of the mutant is as similar as the wild-type protein, indicating that the mutation did not interfere with the overall structures.

Electrophoretic Mobility Shift Assays (EMSA). The DNA segments are obtained from the annealing experiment through the primer 5'-ATATACCATATTATTGCTATTGCCA-3'. Different weights of p35 wild-type protein (10 μg, 20 μg, 30 μg, 40 μg, 50 μg) were incubated individually with the same weight of double-stranded DNA (5 μg) under the binding buffer (20 mM HEPES, 150 mM NaCl, PH 7.5). After incubation at 16°C for 4 hours, 1.5 μl of 10× colourless DNA loading buffer was added. The reaction mixture was then analyzed by 1% agarose gel in 1×TAE buffer (40 mM Tris, 2 mM EDTA), and DNA was visualized by superRed (biosharp) staining.

Liposome floatation experiments. The Liposome floatation experiments were preform as previously described in some modification (Ruigrok et al., 2000). In brief, the Pre-formed liposomes of different PC (1-palmitoyl-2-oleyl-sn-glycero-3-phosphocholine) anneal, PS (1, 2-dioleyl-sn-glycero-3-phospho-L-serine) (Aladdin) composition and cholesterol (Aladdin) was dissolved in chloroform and dried under a nitrogen stream and Vacuum concentrator. Then, resuspended pre-formed liposomes in a HEPES buffer (20 mM HEPES, 150 mM NaCl, pH7.5) were diluted to a final lipid concentration of 10 mM. Large unilamellar vesicles (LUVs) were made by 10 freeze-thaw cycles in liquid nitrogen. LUVs were then passed through a polycarbonate membrane with 100 nm pore size (Avanti Polar Lipids). The types of liposomes used in the investigation were 100 nm PS: PC: cholesterol (10:40:50 mol %) liposomes, 100 nm PS: PC: cholesterol (25:25:50 mol %) liposomes, 100 nm PS: PC: cholesterol (25:25:50 mol %) liposomes). Liposome association was assayed using a discontinuous Accudenz gradient. Different Liposomes (1 mM lipid concentration) were separately incubated with p35 wild-type protein or mutant protein (R16A+K168A++R244A+R278A) at a 100 Lipid: Protein molar ratio in the HEPES buffer overnight at 4℃. After incubation, 225 μl of the protein/liposome mixture was added to 225 μl 80% (w/v) Nycodenz

solution, bringing it to a 40% (w/v) Nycodenz solution. The mixture was placed into a 5x41 mm Ultra-Clear tube (Beckman) overlaid with 375 μl 30% (w/v) Nycodenz solution in the HEPES buffer and then covered with 75 μl HEPES buffer without glycerol. The gradient was centrifuged in an Sw-55 Ti rotor for 5 hours at 48,000 rpm at 4℃. A total of nine fractions were collected from the top to the bottom of the centrifuge tube, each with a volume of 100 μl. The fraction samples were analyzed on a 12% SDS-PAGE and stained with a rapid silver staining kit.

Data accessibility. Atomic coordinates and structure factors for the protein of ASFV p35 has been deposited in the Protein Data Bank under accession number 7BQA.

Reference

Adams, P.D., Grosse-Kunstleve, R.W., Hung, L.-W., Ioerger, T.R., McCoy, A.J., Moriarty, N.W., Read, R.J., Sacchettini, J.C., Sauter, N.K., and Terwilliger, T.C. (2002). PHENIX: building new software for automated crystallographic structure determination. Acta Crystallographica Section D Biological Crystallography 58, 1948-1954.

Chen, V.B., Arendall, W.B., Headd, J.J., Keedy, D.A., Immormino, R.M., Kapral, G.J., Murray, L.W., Richardson, J.S., and Richardson, D.C. (2009). MolProbity: all-atom structure validation for macromolecular crystallography. Acta Crystallographica Section D Biological Crystallography 66, 12-21.

Emsley, P., and Cowtan, K. (2004). Coot: model-building tools for molecular graphics. Acta Crystallographica Section D Biological Crystallography 60, 2126-2132.

Reifschneider, N.H., Goto, S., Nakamoto, H., Takahashi, R., Sugawa, M., Dencher, N.A., and Krause, F. (2006). Defining the Mitochondrial Proteomes from Five Rat Organs in a Physiologically Significant Context Using 2D Blue-Native/SDS-PAGE. Journal of Proteome Research 5, 1117-1132.

Ruigrok, R.W.H., Barge, A., Durrer, P., Brunner, J., Ma, K., and Whittaker, G.R. (2000). Membrane Interaction of Influenza Virus M1 Protein. Virology 267, 289-298.

TABLE 1 Data collection and refinement statistics *

Parameter	p35 (K_2HgI_4)	p35 (native)
PDB accession no.		7BQA
Crystallization pH	7.0	5.0
X-ray source	SSRF BL19U1	SSRF BL17U1
Wavelength (Å)	0.9798	1.0083
Space group	*P*21	*P*21
Unit cell parameters (Å; °)	a=49.348,b=65.444,c= 57.616; α=γ=90, β= 93.88	a=58.068,b=89.563,c = 65.080; α=γ=90, β= 89.95
Resolution range (Å)	30.00-2.70 (2.75-2.70)	50.00-2.10(2.14-2.10)
No. of unique reflections	19625 (882)	37964 (1940)
Completeness (%)	98.3 (88.4)	97.8 (99.8)
Redundancy	3.3 (2.8)	5.8 (6.7)
I/σ(I)	15.8 (2.6)	4.0 (4.1)
Rmerge (%)	7.9 (26.7)	10.6 (54.7)
CC1/2	0.99 (0.92)	0.97 (0.86)
**Refinement statistics**		
Resolution range (Å)		44.78-2.10(2.18-2.10)
No. of reflections used in refinement		37032 (3437)
No. of reflections used for Rfree		1772 (153)
R_{work} (%)		20.3 (24.1)
R_{free} (%)		23.0 (29.7)
No. of nonhydrogen atoms		4663
Protein		4405
Solvent		258
Avg B-factors		26.98
Protein		26.42
Solvent		36.67
RMSD		
Bond length (Å)		0.004
Bond angle (°)		0.97
MolProbity clash score		8.86
Ramachandran (%)		
Favoured		95.42
Allowed		4.58
Outliers		0.00

*Numbers in the brackets are for the highest resolution shell. $R_{merge} = \Sigma_h\Sigma_I \mid I_{ih} - \langle I_h \rangle \mid / \Sigma_h\Sigma_I \langle I_h \rangle$, where $\langle I_h \rangle$ is the mean of the observations I_{ih} of reflection h. $R_{work} = \Sigma(\|F_p(obs)\|-\|F_p(calc)\|)/\Sigma\|F_p(obs)\|$; R_{free} is an R factor for a pre-selected subset (5%) of reflections that was not included in refinement.
